# The African turquoise killifish: A research organism to study vertebrate aging and diapause

**DOI:** 10.1111/acel.12757

**Published:** 2018-03-24

**Authors:** Chi‐Kuo Hu, Anne Brunet

**Affiliations:** ^1^ Department of Genetics Stanford University Stanford CA USA; ^2^ Glenn Laboratories for the Biology of Aging Stanford CA USA

**Keywords:** accelerated aging, aging, anti‐aging, diapause, killifish, rejuvenation

## Abstract

The African turquoise killifish has recently gained significant traction as a new research organism in the aging field. Our understanding of aging has strongly benefited from canonical research organisms—yeast, *C. elegans*,* Drosophila*, zebrafish, and mice. Many characteristics that are essential to understand aging—for example, the adaptive immune system or the hypothalamo‐pituitary axis—are only present in vertebrates (zebrafish and mice). However, zebrafish and mice live more than 3 years and their relatively long lifespans are not compatible with high‐throughput studies. Therefore, the turquoise killifish, a vertebrate with a naturally compressed lifespan of only 4–6 months, fills an essential gap to understand aging. With a recently developed genomic and genetic toolkit, the turquoise killifish not only provides practical advantages for lifespan and longitudinal experiments, but also allows more systematic characterizations of the interplay between genetics and environment during vertebrate aging. Interestingly, the turquoise killifish can also enter a long‐term dormant state during development called diapause. Killifish embryos in diapause already have some organs and tissues, and they can last in this state for years, exhibiting exceptional resistance to stress and to damages due to the passage of time. Understanding the diapause state could give new insights into strategies to prevent the damage caused by aging and to better preserve organs, tissues, and cells. Thus, the African turquoise killifish brings two interesting aspects to the aging field—a compressed lifespan and a long‐term resistant diapause state, both of which should spark new discoveries in the field.

## 1 RESEARCH ORGANISMS FOR AGING

Aging is one of the greatest risk factors for many diseases, and it ultimately restricts the lifespan of organisms by increasing the probability of death (Dillin, Gottschling & Nystrom, 2014; Guarente, Ruvkun & Amasino, 1998; Kennedy et al., 2014; Kenyon, 2005; Niccoli & Partridge, 2012). Since the discovery and characterization of the first long‐lived mutants in *Caenorhabditis elegans* and *Saccharomyces cerevisiae* more than two decades ago (Kaeberlein, McVey & Guarente, 1999; Kenyon, Chang, Gensch, Rudner & Tabtiang, 1993; Morris, Tissenbaum & Ruvkun, 1996; Ogg et al., 1997; Wang et al., 1993), the concept that aging is a malleable biological process has been well embraced (Finch & Ruvkun, 2001; Gems & Partridge, 2013; Kennedy, 2008; Kenyon, 2005, 2010).

However, many mysteries about aging remain. While virtually all species experience aging, organismal lifespan is an amazingly diverse trait in nature. For example, the documented maximal lifespans range from days in the medfly to over 500 years in clams, comprising all intermediaries including ~4 months in fruit flies, ~4 years in mice, ~120 years in humans, ~150 years in giant tortoises, and ~400 years in Greenland sharks (Butler, Wanamaker, Scourse, Richardson & Reynolds, 2013; Castanet, 1994; Karney et al., 2011; Luckinbill & Clare, 1985; Miller, Harper, Dysko, Durkee & Austad, 2002; Nielsen et al., 2016). The extraordinary diversity of lifespan and life history of various organisms raises many exciting possibilities for modeling and understanding the aging process. The lowering costs of genome sequencing and the emergence of cutting‐edge genome‐editing technologies such as CRISPR/Cas9 have enabled the development of virtually any organism into a tractable research system (Sanchez Alvarado, 2018). This development will allow the aging field not to be limited by the specific features of canonical research organisms (yeast, *C. elegans*,* Drosophila*, zebrafish and mice) and to exploit the amazing diversity of aging and lifespan traits in nature.

**Figure 1:**
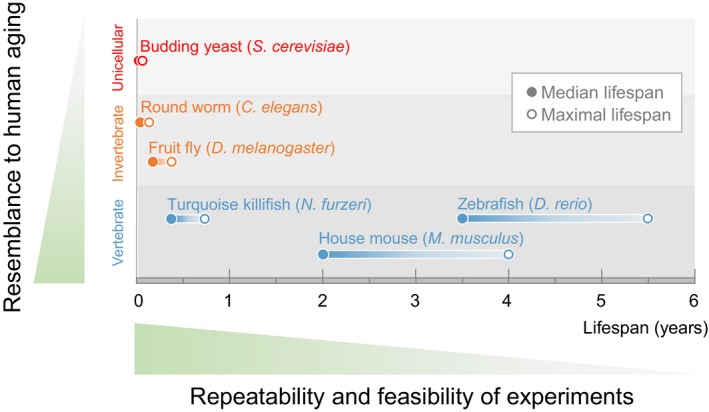
The turquoise killifish is one of the shortest lived, if not the shortest lived, vertebrate. The turquoise killifish is a vertebrate organism with a lifespan closer to that of unicellular and invertebrate canonical research organisms (yeast, round worm, and fruit fly) than vertebrate canonical research organisms (house mouse and zebrafish). The lifespan of each organism is shown with both median (closed circles) and maximal lifespan (open circles). The turquoise killifish, as one of the shortest lived if not the shortest lived vertebrate, is in the unique position to recapitulate some human aging traits and to allow high repeatability and feasibility for experiments

**Figure 2:**
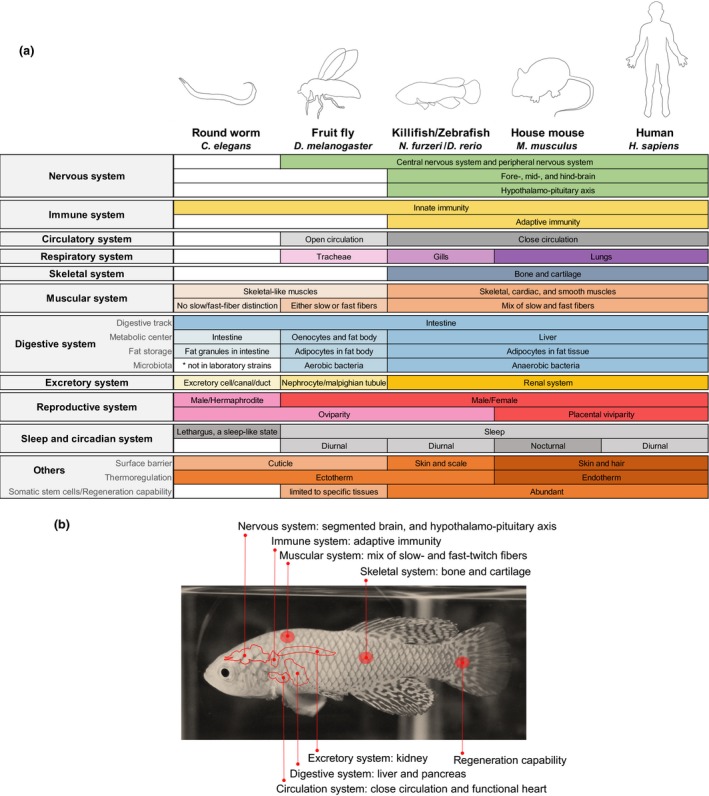
The turquoise killifish contains many biological features of human that are missing in invertebrate canonical research organisms. (a) Comparisons of the main biological systems in the turquoise killifish, human, invertebrate, and vertebrate canonical research organisms. (b) Selected features of the turquoise killifish that are conserved in human and vertebrate canonical research organisms (zebrafish and house mouse), but are missing in invertebrate canonical research organisms (round worm and fruit fly). References: nervous system: Shimeld and Holland (2000), Freeman and Doherty (2006); Lohr and Hammerschmidt (2011), Oikonomou and Shaham (2011), immune system: Langenau and Zon (2005), Engelmann and Pujol (2010), Buchon, Silverman and Cherry *(* 2014); circulatory system: Lehmacher, Abeln and Paululat *(* 2012); Stephenson, Adams and Vaccarezza *(* 2017); respiratory system: Schottenfeld, Song and Ghabrial *(* 2010); skeletal system: Shimeld and Holland (2000); muscular system: Moerman and Williams (2006), Demontis, Piccirillo, Goldberg and Perrimon *(* 2013), Piccirillo, Demontis, Perrimon and Goldberg *(* 2014), Goody, Carter, Kilroy, Maves and Henry *(* 2017); digestive system: McKay, McKay, Avery and Graff *(* 2003), Arrese and Soulages (2010), Hashmi et al. *(* 2013), Kuraishi, Hori and Kurata *(* 2013), Lemaitre and Miguel‐Aliaga (2013), McGhee (2013), Ritter et al. *(* 2013), Berg et al. *(* 2016), Martino, Ma and Leulier *(* 2017), Smith et al. *(* 2017), Tropini, Earle, Huang and Sonnenburg *(* 2017); excretory system: King and Goldstein (1985), Buechner (2002), Gautam, Verma and Tapadia *(* 2017); sleep and circadian system: Raizen et al. *(* 2008); Trojanowski and Raizen (2016); Miyazaki, Liu and Hayashi *(* 2017); others: Micchelli and Perrimon (2006), Ohlstein and Spradling (2006), Chaturvedi, Reichert, Gunage and VijayRaghavan *(* 2017), Gunage, Dhanyasi, Reichert and VijayRaghavan (2017)

As the number of species with emerging interest for research increases (Russell et al., 2017; Sanchez Alvarado, 2018), identifying a system advantageous to answer specific questions is more critical than ever. As laid out by Steve Austad in 1997, choosing a research organism involves tradeoffs between factors such as *realism*,* repeatability of results*, and *feasibility* (Austad, 1997; Figure 1). For years, the aging field has greatly benefited from using short‐lived unicellular organisms (e.g., *S. cerevisiae*) and invertebrates (e.g., *C. elegans* and *Drosophila*) to understand the biological mechanism of aging (Fabrizio & Longo, 2003; Helfand & Rogina, 2003; Kaeberlein, Burtner & Kennedy, 2007; Laun, Rinnerthaler, Bogengruber, Heeren & Breitenbach, 2006; Olsen, Vantipalli & Lithgow, 2006). However, the aging process is considerably more complex in vertebrates. While some key aging genes and pathways are evolutionarily conserved, many systems are vertebrate‐specific (Figure 2). For example, vertebrates have a more specialized nervous system which is divided into a distinct central nervous system that consists of a segmented brain and spinal cord, and a peripheral nervous system that contains ganglia and nerves. These two systems are targeted by different age‐related neural degenerative diseases (e.g., Alzheimer's disease in the central nervous system versus amyotrophic lateral sclerosis in the peripheral nervous system) (Niccoli, Partridge & Isaacs, 2017; Wenk, 2003; Zarei et al., 2015). Vertebrates also have a hypothalamo‐pituitary axis, which is critical for neuro‐endocrine signaling during aging (Veldhuis, 2008). Unlike invertebrates, which rely on an innate immune system, vertebrates have also evolved an adaptive immune system, which plays roles in vertebrate aging in many aspects such as “immunosenescence” (gradual deterioration of the immune system during aging; Weng, 2006; Weyand & Goronzy, 2016) and “inflammaging” (chronic inflammation that occurs during aging; Monti, Ostan, Borelli, Castellani & Franceschi, 2016). Furthermore, aging in vertebrates is often accompanied by the development of cancers (Campisi, 2013), perhaps due to the presence of tissue‐specific stem cells which are more at risk for tumor‐inducing mutation (Batlle & Clevers, 2017). For example, more than half of the newly diagnosed cancers in humans are found in individuals over 65 years of age (Tariman, 2009). This is not the case in worms and flies, which are mostly composed of postmitotic cells. Therefore, studying aging solely in invertebrates does not faithfully reflect all aspects of vertebrate aging, and this lack of “realism” is a tradeoff that hampers our complete understanding of aging. On the other hand, canonical vertebrate research organisms have significantly longer lifespans compared to their invertebrate counterparts. For example, mice and zebrafish have the maximal lifespans of 4 years and 5.5 years, respectively, while *C. elegans* and *Drosophila* have the maximal lifespans of only 4–5 weeks and 3–4 months (Table 1 and Figure 1). The relative long lifespans of mice and zebrafish create an obvious experimental hurdle, limiting both “repeatability of results” and “feasibility.” This tradeoff sacrifices the ability to rapidly and systematically identify, characterize, and verify novel aging genes and networks in vertebrates. As a result, our knowledge of vertebrate aging has remained limited.

**Table 1:** Median and maximal lifespans of the turquoise killifish and other canonical research organisms

Organism		~ Median lifespan	~ Maximal lifespan	References
Budding yeast (*Saccharomyces cerevisiae*)	Unicellular	23 generations	37 generations	Replicative lifespan (Sinclair & Guarente, 1997)
Round worm (*Caenorhabditis elegans*)	Invertebrate	2–3 weeks	4–5 weeks	Bansal, Zhu, Yen & Tissenbaum (2015), Stroustrup et al. (2013) and Sutphin & Kaeberlein, (2009)
Fruit fly (*Drosophila melanogaster*)	Invertebrate	2–2.5 months	3–4 months	Linford, Bilgir, Ro & Pletcher, (2013) and Luckinbill & Clare (1985)
Turquoise killifish (GRZ strain) (*Nothobranchius furzeri*)	Vertebrate	4–6 months	7–9 months	Polacik et al. (2016), Reichwald et al. (2015) and Valenzano et al. (2015)
House mouse (*Mus musculus*)	Vertebrate	2 years	4 years	Miller et al., (2002) and Yuan et al. (2009)
Zebrafish (*Danio rerio*)	Vertebrate	3.5 years	5.5 years	Gerhard et al., (2002) and Keller & Murtha, (2004)

To fill this gap, we and others have actively established the African turquoise killifish *Nothobranchius furzeri* as a research organism for vertebrate aging (Harel, Valenzano & Brunet, 2016; Polacik, Blazek & Reichard, 2016; Reichwald et al., 2015; Valdesalici & Cellerino, 2003; Valenzano, Sharp & Brunet, 2011; Valenzano et al., 2009, 2015). The turquoise killifish experiences a naturally fast‐aging process as part of its natural life history and is the shortest lived vertebrate that can be bred in captivity (Valdesalici & Cellerino, 2003). In addition, along with its short lifespan, the turquoise killifish has another unique but less studied feature called “diapause,” a long‐term dormant state which is resistant to a variety of stresses and to the damages due to the passage of time. In this review, we will discuss the appealing features of the African turquoise killifish as a new vertebrate system while highlighting its contribution within the aging field to date. Our goal is to familiarize the research community with the turquoise killifish and explore its specific niche to advance aging research.

## 2 ESTABLISHING THE AFRICAN TURQUOISE KILLIFISH AS A RESEARCH ORGANISM FOR AGING

First collected and identified as a new species in 1969, the African turquoise killifish is native to East Africa, where it is found exclusively in Zimbabwe and Mozambique (Furzer, 1969; Jubb, 1971; Parle, 1970). While its short‐lived nature was broadly known among killifish hobbyists, the turquoise killifish remained unnoticed to the research community for decades. That was until 2003, when Alessandro Cellerino's group at the Scuola Normale Superiore, in Pisa, Italy, first proposed to use it as a research organism for aging and published its first lifespan of ~2 months (Valdesalici & Cellerino, 2003). Since then, the increased knowledge and improved husbandry have significantly lowered the early mortality rate in captivity. The median lifespan has been thus gradually extended and eventually stabilized at around 4–6 months (Figure 1), with similar numbers reported by multiple institutes across North America and Europe (Polacik et al., 2016; Reichwald et al., 2015; Valenzano et al., 2015).

With years of efforts, the turquoise killifish has been transformed from an animal raised by hobbyists alone into a promising research organism. Critical resources such as standardized and diverse strains, rich genomic information, and powerful toolkits have all been developed. Protocols for easily breeding and rearing the turquoise killifish fish have also been established. These accomplishments have not only transformed the turquoise killifish into a research organism, but they have also eased the process for researchers to integrate this fish into their scientific program.

### 2.1 Resources and tools for the African turquoise killifish

As an organism for research, a critical feature is to have a standardized strain, which helps compare and reproduce experimental results from laboratories over different places and time. The turquoise killifish strain that is most frequently used is the GRZ strain, an inbred line that originates from a sample collected in 1970 in the Gona‐Re‐Zhou National Park of Zimbabwe (Parle, 1970). Since then, several additional samples have been collected in Mozambique and Zimbabwe, and wild strains or strains derived from these samples (e.g., the MZM0403 and 0410 strains which were collected in 2004) have also been broadly used for a variety of studies (Bartakova et al., 2013; Baumgart et al., 2016; Blazek et al., 2017; Kirschner et al., 2012; Reichard, Polacik & Sedlacek, 2009; Reichwald et al., 2015; Terzibasi et al., 2008; Valenzano et al., 2009, 2015). In particular, these strains were used to identify the genetic architecture underlying phenotypic differences in these strains, such as color (Valenzano et al., 2009) and survival under specific laboratory environments (Baumgart et al., 2016; Blazek et al., 2017; Reichwald et al., 2015; Valenzano et al., 2015). Our laboratory has also collected a series of strains through a collection trip to different areas of Mozambique and Zimbabwe in 2010 (ZMZ1001 to 1007). Many of them have already reached the status of inbred lines (>20 single‐paired sibling crossings [C.‐K. Hu and A. Brunet, *unpublished data*]), providing the community with additional options of genetic backgrounds thereby minimizing the risk of the strain‐specific artifacts.

The turquoise killifish reached another critical milestone when its genome was sequenced. In 2015, two independent groups de novo assembled and annotated the turquoise killifish genome: Anne Brunet's laboratory at Stanford University (African Turquoise Killifish Genome Browser: http://africanturquoisekillifishbrowser.org/; Valenzano et al., 2015) and Matthias Platzer's laboratory in Jena, Germany (*Nothobranchius furzeri* Genome Browser: http://nfingb.leibniz-fli.de/; Reichwald et al., 2015). Additionally, other genomic resources have also been established through the years, including genetic linkage maps, quantitative trait loci, over 150 microsatellite markers (Blazek et al., 2017; Kirschner et al., 2012; Valenzano et al., 2009), and numerous transcriptomic and epigenomic datasets of different tissues at different ages using various strains (Baumgart et al., 2012, 2014, 2016, 2017; D'Angelo et al., 2014; Ng'oma, Groth, Ripa, Platzer & Cellerino, 2014; Petzold et al., 2013). A reference genome from the NCBI pipeline was made available online in 2016 (NCBI Genome ID: 2642, URL: https://www.ncbi.nlm.nih.gov/genome/2642). This NCBI reference genome re‐annotated the assembled sequences from the Platzer group by integrating additional information of publicly available omics resources. As each reference genome and derived gene models have their own strengths and advantages, an effort to integrate both reference genomes and their derivative gene models is currently ongoing.

**Figure 3:**
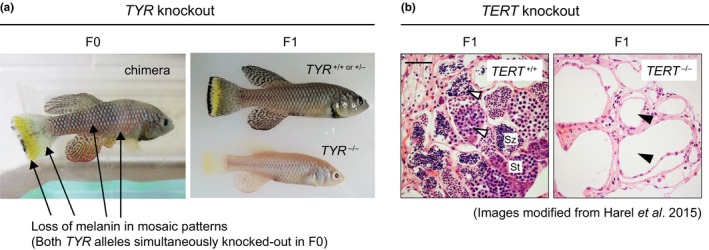
The turquoise killifish shows high CRISPR/Cas9 genome‐editing efficiency (a) *TYR* (Tyrosinase), an essential gene for converting tyrosine to melanin (black pigment), can be used to visualize the efficiency of CRISPR/Cas9 genome‐editing in the turquoise killifish. Melanin is absent in cells when both alleles of the *TYR* gene are knocked out. In F0 fish, large melanin‐negative areas are observed in mosaic patterns, indicating that CRISPR/Cas9 genome‐editing works with high efficiency to simultaneously disrupt both *TYR* alleles in the same cells. Germline transmission is confirmed in F1 offspring where melanin‐positive and melanin‐negative phenotypes in whole fish can be separated. (b) Histological sections of testis from 4 months old F1 *TERT* ^+/+^ and *TERT* ^−/−^ fish generated by CRISPR/Cas9 editing. While germ cells are present in the testis of TERT ^+/+^ fish (white arrowheads), they are severely deficient in the testis of *TERT* ^−/−^ fish (black arrowheads). Sz, spermatozoa (mature sperm); St, spermatids. Scale bar, 50 μm

Great progress has also been made in developing genomic and genetic tools for this fish (Harel et al., 2016; Platzer & Englert, 2016). Transposase Tol2‐based transgenesis was successfully developed in turquoise killifish (Allard, Kamei & Duan, 2013; Hartmann & Englert, 2012; Valenzano et al., 2011). Importantly, the Brunet laboratory at Stanford recently developed a highly efficient CRISPR‐Cas9 genome‐editing pipeline, which allows both knockout and knockin in this fish with very high efficiency (Harel et al., 2015; Figure 3). Given the short lifecycle of the turquoise killifish, a stable line with the desired modification can be generated in 2 to 3 months (Harel et al., 2015), which is faster and more advantageous than both zebrafish and mice especially when the time for back‐crossing is also considered.

### 2.2 Standardized housing and caring for the turquoise killifish

The protocols for housing and caring for the turquoise killifish have been well established and standardized (Harel et al., 2016; Polacik et al., 2016). This significantly facilitates the process of starting the turquoise killifish as a research organism. Establishing turquoise killifish colonies can be relatively easily performed, and it is compatible with various levels of resource and budgets.

The turquoise killifish is easy to raise and can live with a broad range of environmental conditions such as temperature and salinity (Harel et al., 2016; Polacik et al., 2016). Both commercially available systems and homemade racks for zebrafish can be converted for the turquoise killifish, without the need to add specific equipment and devices. The turquoise killifish has a XY sex‐determination system and shows sexual dimorphism after maturation (Reichwald et al., 2015; Valenzano et al., 2009). Males and females are roughly even‐numbered in offspring and are easily distinguished especially when they reach sexual maturity, as males are bigger in size and have colorful fins. Unlike zebrafish, the turquoise killifish does not require specific conditioning to trigger mating and breeding behaviors. Male and female fish can be housed in the same tank and breed continuously, which minimizes the burden of maintenance. While the fecundity declines with age, one breeding pair generally produces tens to hundreds of embryos per week throughout their lives (Polacik et al., 2016; Vrtilek, Polacik & Reichard, 2017). However, it is also important to note that the hatching process of embryos is not automatic and is induced by specific environmental cues. This is different to most teleost embryos (including zebrafish), and it is usually the part of the husbandry protocol that requires the most attention when learning how to establish the killifish in one's own facilities. In addition, unlike zebrafish, killifish males exhibit territorial behaviors and often need to be separated from each other (although they can be housed with females), and this can increase the number of tanks for colony maintenance or lifespan studies.

Together, the diverse inbred lines and wild strains, the existence of two complementary well‐annotated genomes, the ability to manipulate the genome to delete, modify, or overexpress genes, and the straightforward protocol to establish the turquoise killifish colonies in captivity have established this species as a highly promising and easily adaptable system for aging research.

## 3 THE AFRICAN TURQUOISE KILLIFISH LIFECYCLE IS COMPOSED OF TWO DISTINCT PHASES WITH OPPOSING AGING FEATURES

**Figure 4:**
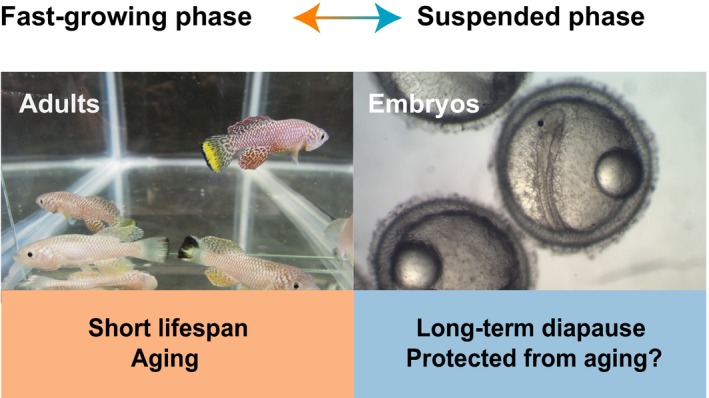
The turquoise killifish has a lifecycle composed of two distinct phases with opposite aging features. After hatching, the turquoise killifish grows fast, reaches sexual maturation fast, and ages fast. This fast‐growing phase occurs during the rainy season of its natural habitat to ensure the turquoise killifish can survive as a species by propagating quickly while water is present. The newly laid embryos can enter diapause to suspend their development during the dry season. The embryos can stay in diapause for months, even several times longer than the fish lifespan, suggesting that the fast‐aging process exhibited during adulthood is blocked during diapause. The embryos then break diapause, and resume their compressed lifecycle in the following rainy season. Some embryos naturally escape diapause, and it is therefore possible to study each state separately. This two‐phased lifecycle is still present in the laboratory with constant water, indicating a genetic underlying

The turquoise killifish is also particularly interesting in that it has two distinct phases with opposing features: (i) a naturally compressed lifespan, with rapid development, sexual maturation, and aging; and (ii) a diapause state, which is a long‐lived form of resistance to a variety of stresses and to damages due to the passage of time. These unique features are evolutionary adaptation to its extreme habitat (Blazek, Polacik & Reichard, 2013). The turquoise killifish naturally live in ephemeral ponds in Mozambique and Zimbabwe where water is only present during the brief rainy season. The rainy season is followed by a prolonged dry season, during which the ponds entirely dry up (Bartakova et al., 2013; Furzer, 1969; Jubb, 1969, 1971; Reichard et al., 2009). This extreme habitat challenges the inhabiting species not only to propagate fast within the short rainy season, but also to survive through the following long dry season. The turquoise killifish has adapted, overevolutionary times, to this extreme environment by having a life history composed of two distinct phases—a fast‐growing (and fast‐aging) phase in the rainy season and then a suspended (and long‐lived) phase in the dry season (Bartakova et al., 2013; Blazek et al., 2013; Reichard et al., 2009; Figure 4). Importantly, these two phases remain unchanged in captivity, even in constant water (Polacik et al., 2016), indicating that both are under genetic determination.

### 3.1 Fast aging in the adult killifish

The fast‐growing phase allows the turquoise killifish to compress its natural lifecycle within the short window of the rainy season (which lasts about 4 months), taking only ~40 days from embryos proceeding to embryos of the next generation (Kim, Nam & Valenzano, 2016; Valenzano et al., 2015). To achieve this compressed lifecycle, the turquoise killifish grows fast, reproduces fast, and, likely as a pleiotropic consequence of these constraints, also ages fast. It can reach sexual maturity and start breeding within 3–4 weeks after hatching. In optimal laboratory conditions, the GRZ strain of the turquoise killifish has a median lifespan of 4–6 months and a maximal lifespan of 7–9 months (Polacik et al., 2016; Reichwald et al., 2015; Valdesalici & Cellerino, 2003; Valenzano et al., 2015; and C.‐K. Hu and A. Brunet, *unpublished data*; Table 1 and Figure 1), which is about 6–7 times shorter than mice and 10–12 times shorter than zebrafish.

**Figure 5:**
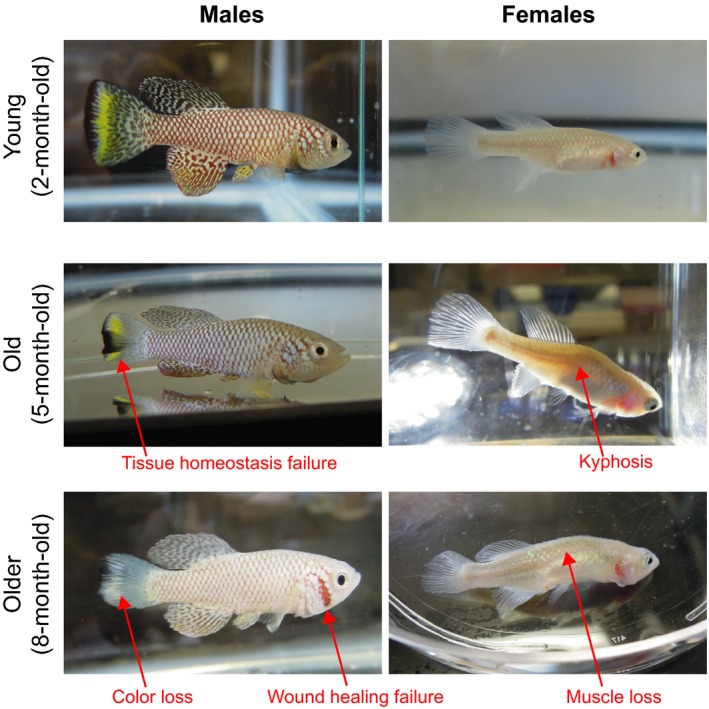
The turquoise killifish shows canonical vertebrate aging phenotypes at the end of its short lifespan. Old fish exhibit obvious outward aging phenotypes in both males and females. Representative stereotypical phenotypes of aging are shown in fish at median lifespan (5 months) and maximal lifespan (8 months). Both males and females are shown. Older fish are paler, exhibit tissue homeostasis defects (e.g., loss of color patterns on fins), fail to properly heal wounds, display spine bending (kyphosis phenotype), and exhibit muscle loss (e.g., muscle loss in the dorsal region and difficulty to swim properly)

Aging in the turquoise killifish has been extensively characterized at both the phenotypical and molecular level in both the GRZ strain and other strains such as MZM0403 and 0410 (Baumgart et al., 2012, 2014; Di Cicco, Tozzini, Rossi & Cellerino, 2011; Hartmann et al., 2009, 2011; Priami et al., 2015; Terzibasi Tozzini et al., 2014; Terzibasi et al., 2008; Tozzini, Baumgart, Battistoni & Cellerino, 2012). Indeed, despite their short lifespan compared to other vertebrates, various strains of the turquoise killifish recapitulate numerous stereotypical aging traits that have been reported in other vertebrates (Figure 5), including decline in reproduction, fertility, cognition, mobility, regeneration, and tissue homeostasis, along with increased incidence of senescence, neural and muscular degeneration, and cancerous lesions (Di Cicco et al., 2011; Terzibasi, Valenzano & Cellerino, 2007; Terzibasi et al., 2008; Valenzano, Terzibasi, Cattaneo, Domenici & Cellerino, 2006; Wendler, Hartmann, Hoppe & Englert, 2015). Importantly, the turquoise killifish does not die of just one disease, but appears to have multiple causes of death in old age, indicating that its lifespan is truly compressed rather than limited by a specific disease. Old turquoise killifish also exhibit molecular markers of aging, such as a decrease in mitochondrial DNA copy number and telomere length with age (Hartmann et al., 2009, 2011). Finally, environmental stimuli known to impact lifespan in other species (e.g., dietary restriction) also extend the lifespan of the turquoise killifish (Terzibasi et al., 2009). Thus, the turquoise killifish is likely to provide an adequate research system for studying vertebrate aging at both physiological level and molecular level, and indeed, it has been used by several groups in this capacity, for example, to uncover the role of mitochondrial genes in predicting remaining lifespan (Baumgart et al., 2016).

### 3.2 Resistance of aging in diapause embryos

The suspended, long‐lived phase of the turquoise killifish lifecycle takes place at the embryonic stage. Newly laid embryos quickly enter a dormant state called “diapause,” which suspends all developmental processes and provides higher tolerance to various stresses, including the long drought. The diapause embryos remain in this suspended state until the next rainy season, where the ponds refills the ponds with water again. The dry season usually lasts about 6–8 months, but can also extend to years (Reichard et al., 2009). Remarkably, the time in diapause can exceed by several times the median lifespan of the turquoise killifish (C.‐K. Hu and A. Brunet, *unpublished data*), suggesting that during diapause this species does not experience the same fast “aging clock” as in the rainy season. The accumulation of damage from the passage of time is likely blocked or attenuated in this dormant state. Importantly, while the majority of embryos obligatorily enter diapause, some embryos naturally skip diapause and exhibit a continuous lifecycle, probably as a hedge‐betting evolutionary mechanism (Polacik et al., 2014; Furness, Lee & Reznick, 2015, and C.‐K. Hu and A. Brunet, *unpublished data*). Diapause and nondiapause embryos provide a set of potent experimental materials to gain insight into not only the regulation of diapause but also the changes in the aging process during and beyond diapause.

The existence of both diapause and nondiapause populations at the embryonic stage is also advantageous for managing animals for research. On the one hand, diapause provides a convenient way to store precious strains long‐term without the hassle of continuous maintenance. On the other hand, nondiapause embryos also allow the turquoise killifish colonies to be continuously maintained or quickly expanded in captivity without the extra hurdle of diapause.

## 4 USING THE AFRICAN TURQUOISE KILLIFISH AS A RESEARCH ORGANISM FOR AGING

### 4.1 Studying aging with the short lifespan of the turquoise killifish

The short lifespan of the turquoise killifish offers both experimental and practical advantages for aging research. Experimentally, the naturally compressed lifespan provides a great platform to systematically understand the aging process and dissect the regulatory mechanisms of vertebrate aging. Practically, the short lifespan of the turquoise killifish makes it feasible to perform multiple rounds of hypothesis‐driven lifespan experiments within the course of a grant or the average training period for graduate students or postdocs. The relatively low cost of maintaining a large cohort of turquoise killifish also makes this organism an appealing vertebrate research system for high‐throughput screens for lifespan or aging.

#### 4.1.1 An easy vertebrate system to study lifespan, healthspan, and aging

One immediate value that the turquoise killifish has for aging research is to complement the longer lived well‐established research organisms. The turquoise killifish, as a short‐lived vertebrate, is in the sweet spot between traditional invertebrate and vertebrate research systems (Figure 1). On the one hand, it is an ideal vertebrate platform to test candidates from invertebrates such as *C. elegans* or *Drosophila*. On the other hand, it can also be employed as a fast‐tracked pipeline to functionally verify discoveries from longer lived vertebrates such as mice, humans, and long‐lived species (bowhead whale, naked mole rat, elephant, etc.).

A common strategy in aging research is to collect data at a few age points across the lifespan and reconstruct the aging process from these snapshots (Golden & Melov, 2007; Rogers, 1991). This scheme has proven successful in short‐lived invertebrate organisms, in which a few age points are sufficient to provide information continuity throughout lifespan (Golden & Melov, 2007). However, in canonical vertebrate research organisms such as mice and zebrafish, it is more difficult to reconstruct the aging process from a few points, because the age points are often too far apart. The turquoise killifish provides a great opportunity to fill this gap, as its compressed lifespan requires fewer data points to capture the kinetics of the aging process (Baumgart et al., 2014). In addition, the turquoise killifish exhibits various complex behaviors such as learning that could be monitored and measured (Valenzano, Terzibasi, Vattaneo et al., 2006a, 2006b). Coupling the compressed aging process with systematic behavior analysis such as cognitive decline adds more information to aging studies.

In addition to complementing canonical research organisms, the turquoise killifish has specific advantages to study the molecular mechanism of aging. As aging is a continuous biological process, longitudinal studies are an integral component of aging research (NASEM 2017). A short lifespan allows rapid systematic longitudinal studies to efficiently correlate molecular mechanisms with lifespan trajectories in vertebrates and identify predictive features of longevity. For example, a longitudinal analysis linked the transcriptomes of caudal fins at different ages in individual fish to the lifespan of the same individuals and showed that the mitochondrial respiratory chain complex I functions as a hub of genes whose expression is negatively correlated with maximal lifespan (Baumgart et al., 2016).

The turquoise killifish's short lifespan provides a favorable scaling between chronological age and aging. For example, while a 3‐month‐old turquoise killifish of the GRZ strain is entering old age (Table 1), a 3‐month‐old zebrafish just reaches sexual maturation to breed (Avdesh et al., 2012). The turquoise killifish thus provides a fast and versatile genotype‐to‐phenotype platform, which is especially helpful for studying aging‐related diseases, including cancer and neurodegeneration (Di Cicco et al., 2011; Harel et al., 2015; Terzibasi Tozzini et al., 2014; Tozzini et al., 2012). A good example is the telomerase reverse transcriptase (*TERT*), which when mutated in the turquoise killifish, results in phenotypes that are similar to those seen in humans deficient for telomerase: defects in actively proliferating organs and systems, such as blood, gut, and gonads (Harel et al., 2015; Figure 3). Importantly, the telomeres in the turquoise killifish are more comparable in length to human telomeres (~8 kb) than mouse telomeres (which are extremely long ~50–150 kb; Harel et al., 2015; Hartmann et al., 2009), making this fish a great research system to model and study telomere attrition in the context of aging and telomere‐related pathologies (e.g., age‐dependent stem cell exhaustion) in vivo.

Finally, the turquoise killifish could also facilitate new areas at the nexus between aging and other fields. For example, the turquoise killifish, with a short lifespan and ability to regenerate tissues, is in a great position to study the interplay between tissue regeneration and aging in vertebrates (Hoppe et al., 2015; Wendler et al., 2015).

#### 4.1.2 A research system to dissect the interplay between genetics and environment during aging

As aging is a complex phenotype regulated by both genetic and environmental factors (Lopez‐Otin, Blasco, Partridge, Serrano & Kroemer, 2013), it is critical to understand the influence of both factors. A common practice to isolate the genetic components of aging is to maintain a constant environment in lifespan experiments. This practice requires extensive monitoring of the environment and constant regulation of all variables, which can be experimentally challenging when the lifespan is long (Solleveld & Hollander, 1984). With the turquoise killifish, the lifespan experiments can be completed within a shorter period of time, therefore minimizing the risk of undesired interference from the environment (Kim et al., 2016). While this practice helps focus on the genetic components of aging, it leaves the environmental inputs of aging and the interplay between genetics and environment insufficiently studied. The turquoise killifish has the potential to fill this long overdue need. The killifish short lifespan makes it feasible to precisely manipulate the environment over the same short experimental cycle. The lifespan of turquoise killifish is responsive to environmental stimuli that affect the rate of aging in virtually all species (e.g., dietary restriction [Terzibasi et al., 2009]), or in some species (e.g., temperature [Valenzano, Terzibasi, Vattaneo et al., 2006a, 2006b] and Resveratrol [Valenzano, Terzibasi, Genade et al., 2006]), making the turquoise killifish a great vertebrate research organism to systematically study the environmental inputs of aging. Its compressed lifecycle and short lifespan allow relatively high‐throughput lifespan experiments, thereby rendering the study of environmental influence, and its interplay with the genetic background, on aging more feasible. A good example is the influence of gut microbiota on lifespan. A recent study using the turquoise killifish to address this question has revealed that not only the gut microbiota dynamically evolves with the host at different ages, but the host's lifespan can also be effectively extended by acquiring microbiota from the younger gut (Smith et al., 2017). The killifish short lifespan plays a key role to allow systematically characterize and manipulate the gut microbiota at different ages, and then assess their impact on the lifespan of the same individuals (Smith et al., 2017). With the turquoise killifish and its compressed lifespan, some burning questions about aging can start to be systematically examined, including the impact of the timing of sexual maturation, the tradeoff between reproduction and lifespan, the role of specific nutrients in regulating aging, and even the importance of social interactions in the aging process.

#### 4.1.3 A unique reference to study the evolution of aging with the unique annual life cycle

The evolution of aging remains one of the biggest unsolved questions in biology, and the turquoise killifish's exceptionally short lifespan grants it a unique position in aging research from an evolutionary perspective. While several evolutionary studies have focused on exceptionally long‐lived animals such as bowhead whale and naked mole rat, the turquoise killifish provides a new perspective from the other side of the lifespan spectrum (“life in the fast lane”; Keane et al., 2015; Reichwald et al., 2015; Valenzano et al., 2015).

The annual lifecycle of the turquoise killifish and many other killifishes, also known as “annualism,” is a textbook example of species evolved under strong selecting pressures to adapt to extreme habitats (Murphy & Collier, 1997). The turquoise killifish rushes to reach sex maturity to propagate within the short rainy season. One critical yet unanswered question is whether the fast‐aging process is also a consequence of the evolution of annualism. If it is, what is the biological tradeoff for this shorter lifespan? Could it be the fast sexual maturation rate and reproduction cycles? And would the same tradeoff be present, albeit “in reverse,” for vertebrates to evolve longer lifespan, such as olm (human fish, *Proteus angeius*) which has an exceptionally long lifespan (predicted maximum lifespan of over 100 years) and also long reproduction cycles (age of sexual maturation at ~16 year, with ~35 eggs every 12.5 years; Voituron, de Fraipont, Issartel, Guillaume & Clobert, 2011)?

While the extremely long‐lived vertebrates are an obvious choice as the out‐group for comparison in evolutionary studies, another good candidate is other killifishes living in constant water without the selecting pressure of the annual drought. Those nonannual killifish do not have the diapause phase and generally have much longer lifespans (Reichwald et al., 2015; Sahm, Platzer & Cellerino, 2016), making them a great resource for comparative studies with the turquoise killifish to tackle the evolution of annualism and its links with aging. Two independent and complementary studies of the positive selection on the turquoise killifish genome have been carried out by the Brunet laboratory and the Englert, Cellerino and Platzer groups, using the long‐lived vertebrates and the nonannual killifish, respectively, as the out‐groups (Reichwald et al., 2015; Sahm et al., 2016; Valenzano et al., 2015). These two approaches focused on different evolutionary scales and identified distinct genes under positive selection in the turquoise killifish genome.

Remarkably, many aging‐related genes are found under the positive selection in these studies, including BAX1, IRS1, IGFR1A, and XRCC5 (Reichwald et al., 2015; Sahm et al., 2016; Valenzano et al., 2015). Interestingly, lamin A (LMNA), which causes progeria in human when carries specific deleterious mutations (De Sandre‐Giovannoli et al., 2003), was also positively selected in the turquoise killifish genome during evolution (Valenzano et al., 2015). However, it is important to note that the turquoise killifish's fast‐aging process is unlikely to be just a pathological consequence of a progeria‐like state. First, this fish exhibit clear neurological aging phenotypes (declining neurogenesis and cognition; Tozzini et al., 2012; Valenzano, Terzibasi, Vattaneo et al., 2006a, 2006b), and those are not seen in progeria patient. Second, the cells from old killifish do not exhibit the irregular shape of nuclei that is characteristic of progeria (C.‐K. Hu and A. Brunet, *unpublished data*). Thus, it is more likely that this fish exhibit a natural variant of lamin A, like some human centenarians do (Lattanzi et al., 2014), perhaps influencing some specific aspects of the physiological aging process. In the future, as various hypotheses have been proposed to explain the evolutionary origin of aging (e.g., Mutation Accumulation, Antagonistic Pleiotropy), the turquoise killifish's short lifespan will allow interesting candidates from the evolutionary genomics studies to be experimentally tested for their role in lifespan and healthspan, which is virtually impossible to do in long‐lived species**.**

### 4.2 Studying diapause, and its link with aging, in the turquoise killifish

While the naturally compressed lifespan of the turquoise killifish has great advantages for studying vertebrate aging, the diapause phase of this fish also provides a unique opportunity to understand states of extreme stress resistance and even “aging resistance.”

Diapause is a specialized state characterized not only by a temporal suspension of development but also by an extreme tolerance to various stresses to protect offspring from an adverse environment (MacRae, 2010; Schiesari & O'Connor, 2013). As a consequence, it helps the species survive extreme forms of stress (e.g., drought) and it also “times” the birth of offspring to a more favorable environment (e.g., rainy season). In nature, diapause phenomena are widespread throughout the animal kingdom, from simple organisms such as brine shrimps and silkworms, to mammals such as the roe deer, bats, and mice (Bleier, 1975; Emerson, Bradshaw & Holzapfel, 2009; Hand, Denlinger, Podrabsky & Roy, 2016; Lambert et al., 2001; Lopes, Desmarais & Murphy, 2004; Meenakumari & Krishna, 2005; Ptak et al., 2012; Sato et al., 2014; Schiesari & O'Connor, 2013; Sim & Denlinger, 2013). Our current knowledge of diapause has benefited greatly from the studies in invertebrates. A well‐documented example is the *C. elegans* diapause (or diapause‐like) state called “dauer,” which helps the species survives a dearth of food (Frezal & Felix, 2015). Notably, although organisms in diapause are developmentally suspended, they remain physiologically active and can even be mobile. For example, *C. elegans* in the dauer state can move long distance searching for new food sources (Frezal & Felix, 2015) and Monarch butterfly in its reproductive diapause form can migrate over 3,000 miles from southern Canada and Midwestern United States to overwintering sites in central Mexico (Tatar & Yin, 2001; Yakubu, Saenz, Stein & Jones, 2004). Invertebrate systems have also revealed organismal and genetic links between diapause and aging. First, the individuals that undergo diapause in *C. elegans* or *Drosophila* resume a normal lifespan when they reach adulthood (Klass & Hirsh, 1976; Kucerova et al., 2016; Zhao et al., 2016). In other documented examples of insects, individuals that have been through diapause significantly even outlive their counterparts that have never entered diapause (Tatar & Yin, 2001). Thus, the aging process appears to be slowed down or even suspended during diapause and diapause embryos appear to be protected from damage that accumulates with the passage of time. Alternatively, some aspects of aging may occur, with some damage accumulating, but these aging hallmarks could be “reset” at the exit from diapause, as has been shown for *C. elegans* (Klass & Hirsh, 1976; Roux, Langhans, Huynh & Kenyon, 2016). Second, it is important to note that, at least in *C. elegans*, the genetic network underlying the dauer state shares many components with the genetic network of aging, with notably a key role for the insulin pathway (Antebi, 2013; Fielenbach & Antebi, 2008; Girard et al., 2007; Padilla & Ladage, 2012).

Thus, studying diapause could not only offer insight into the genetic network that regulates lifespan but also provide new ideas for prevention of damage accumulation or erasure of damage.

The interplay between diapause and aging has been vastly understudied in the context of vertebrate systems, mostly due to the low accessibility of embryos and the difficulty to estimate the impact of diapause over a long lifespan. In mammals, the low number of embryos and their poor accessibility in the womb has significantly hampered the study of diapause, particularly its relationship with aging. The turquoise killifish is well suited for overcoming these obstacles with its high number of transparent embryos outside of the female body and its short lifespan. These features make it easy to observe, obtain, and manipulate the embryos before, during, and after diapause for various types of experiments, including microscopy, biochemistry, and genomics.

Diapause can occur at different developmental stages in diverse species (Hand et al., 2016; Lopes et al., 2004; Mead, 1993; Ptak et al., 2012). In killifishes, diapause can occur during early, intermediate, and late developmental stages (diapause I, II, and III, respectively; Wourms, 1972). Diapause II, which occurs at the intermediate developmental stage, can last longer than diapause I and III, and this is the state that allows killifish species to survive throughout harsh environment (Wourms, 1972). We will refer to diapause II as “diapause” in the remainder of this review. An interesting feature of the turquoise killifish diapause is that it can last longer than the entire adult lifespan (Polacik et al., 2016; C.‐K. Hu and A. Brunet, *unpublished data*). This exaggerated phenotype is very rare in vertebrates that can do diapause, even among killifishes, and it makes the study of the impact of diapause on aging easier. Indeed, while other longer lived killifishes have been historically employed to study diapause, most of these studies have been focused on the stress resistant feature of diapause rather than its “aging resistance” (Meller, Meller, Simon, Culpepper & Podrabsky, 2012; Podrabsky & Hand, 1999, 2000; Woll & Podrabsky, 2017). Thus, the turquoise killifish provides a unique platform to address many critical questions of vertebrate diapause, including if there are shared components between diapause and normal aging (Reichwald et al., 2015), if and how the aging process is blocked during diapause, and if there is any tradeoff between the long‐term survival in diapause and the physiological status in adulthood.

Importantly, studying diapause could also provide new insights into aging interventions and healthspan extension. For example, the collapse of homeostasis is one of the critical features of aging (Frakes & Dillin, 2017; Gervais & Bardin, 2017; Lopez‐Otin et al., 2013).

Understanding how organisms maintain and regulate homeostasis during diapause may reveal new strategies to prevent this collapse during aging. Studying diapause in the turquoise killifish could also provide a novel understanding not only of the molecular mechanisms of homeostasis but also of cellular mechanisms of maintenance in tissues. For example, how are stem cells, progenitor cells, and differentiated cells in tissues and organs protected and maintained throughout this long‐term suspended state? Such knowledge could then be harnessed to help prevent systematic tissue degeneration and diseases during the normal aging process.

## 5 CONCLUSION

As highlighted in this review, the turquoise killifish has emerged as a promising new research organism for vertebrate aging and aging‐related diseases. With its unique features—a naturally compressed lifespan and a suspended developmental state—the turquoise killifish nicely complements the currently used research organisms for a more systematic understanding of aging and longevity.

## CONflICT OF INTEREST

Both authors declare to have no conflict of interest.
